# The Antioxidative Role of Autophagy in Hearing Loss

**DOI:** 10.3389/fnins.2018.01010

**Published:** 2019-01-09

**Authors:** Bin Ye, Cui Fan, Yilin Shen, Quan Wang, Haixia Hu, Mingliang Xiang

**Affiliations:** ^1^Department of Otolaryngology & Head and Neck Surgery, Ruijin Hospital, Shanghai Jiao Tong University School of Medicine, Shanghai, China; ^2^Ear Institute, Shanghai Jiao Tong University School of Medicine, Shanghai, China; ^3^Shanghai Key Laboratory of Translational Medicine on Ear and Nose Diseases, Shanghai, China

**Keywords:** sensorineural hearing loss, autophagic mechanism, oxidative stress, antioxidant, hearing preservation

## Abstract

Autophagy, a highly conserved cellular mechanism, plays an essential role in the development and pathology of many central and peripheral nervous system diseases. The auditory system, especially hair cells (HCs) and spiral ganglion neurons (SGNs) in the inner ear, are postmitotic cells, which are extremely reliant on cellular homeostasis and energy supply. Therefore, autophagy may be involved in contributing to and facilitating the normal function of inner ear cells. Recently, studies on hearing loss induced by ototoxic drugs, noise exposure and other factors have revealed that autophagy could serve in an antioxidative capacity and could possess the potential to treat sensorineural hearing loss (SNHL). Therefore, here we review previous studies concerning autophagy and SNHL to gain insight into the role of autophagic mechanisms in inner ear disorders.

## Introduction

The term “autophagy” originates from Greek and means “self-devouring.” The Belgian scientist Christian René de Duve, who won the Nobel Prize in Physiology or Medicine for his identification of lysosomes and peroxisomes, first proposed the concept of “autophagy.” In eukaryotic cells, autophagy refers to the fact that under conditions of stress such as nutrient starvation, damaged proteins and other components within the cell body are translocated into the lysosome and then degraded into small compounds for reuse, which sustains cellular homeostasis. In short, autophagy is the intracellular process of “self-devouring” and “self-powering.” However, autophagy was not known to the public until the Japanese scientist Yoshinori Ohsumi uncovered a series of critical genes regulating autophagic mechanisms through the study of a starvation-induced yeast model. In recent years, major achievements regarding autophagic mechanisms have been made in neurodegenerative diseases, cardiovascular diseases, tumors and aging, and many researchers have considered that it has great possibilities for application. In the auditory system, especially the inner ear, several studies have noted the relationship between autophagy and animal models of hearing loss and that upregulating the autophagic mechanism contributes to alleviating the morphological damage to the inner ear and subsequent hearing loss. In this paper, we review and discuss the connections between autophagy and hearing loss induced by noise exposure, ototoxic drugs, aging and genetic mutations to determine the specific autophagic mechanism involved in hearing loss, and further discuss whether autophagic mechanisms could serve in potential antioxidative approaches to rescue hearing.

## The Concept and Process of Autophagy

Autophagy is a cellular pro-survival pathway. When cells are under conditions of stress, such as caloric restriction and oxidative stress, abnormal or damaged cytoplasmic components, including abnormal or damaged proteins, lipids and organelles, are contained, engulfed and trafficked into lysosomes, and degraded into small compounds such as amino acids, fatty acids and nucleotides for recycling (Mizushima et al., 2008). There are three types of autophagy based on the various degradation processes: macroautophagy, microautophagy, and chaperone-mediated autophagy. All of these share the feature that all substrates will ultimately be degraded within lysosomes. The term “autophagy” here generally refers to macroautophagy, which is the easiest to observe and best explored type of autophagy. Autophagy is characterized by the formation of a double-membrane autophagosome with a diameter of approximately 1 μm. Briefly speaking, the entire autophagy process includes five stages (Yoshimori, 2004; Mizushima et al., 2008; He and Klionsky, 2009): (1) autophagy initiation: intracellular upstream signaling pathways of autophagy, such as the mTOR and AMPK pathways, are activated in response to external stress, after which the level or transcriptional activity of autophagy-related genes (ATGs) are upregulated, and autophagic mechanisms are initiated; (2) autophagosome precursor formation: under the stimulation of autophagy-inducing signals, autophagy-related proteins such as Beclin-1 and Vps34 are progressively recruited to the surroundings of autophagic cargoes to generate the autophagosome precursors; (3) autophagosome formation: after persistently containing, elongating and engulfing damaged proteins and organelles, the autophagosome precursor evolves into a closed double-membrane structure known as an autophagosome; (4) autolysosome formation: the autophagosome is transported to the lysosome and fuses with it to form the autolysosome; (5) degradation of cargoes: cargoes in the autophagosome are degraded into small molecular materials by lysosomal hydrolases and proteases and finally are released into the cytoplasm for reuse.

## The Role of Autophagy in Hearing Loss

In the auditory field, limited research on autophagy has been reported to date, and most such studies focused on utilizing autophagic mechanisms to treat SNHL caused by specific factors, especially HC damage. Here, to better understand the relationship between autophagy and hearing loss, we briefly summarize the advances in understanding from studies of autophagy in inner ear development and hearing loss.

### Autophagy Participates in Normal Cochlear Development

The inner ear is a sensory organ characterized by distinct intracellular heterogeneity and precise spatial organization. Autophagy, modulated by growth factor signaling, contributes to cellular differentiation by providing energy and materials (Magarinos et al., 2017). A previous study using real-time PCR found that *ATG4b*, *ATG5*, *ATG9a,* and *Becn1* levels in the mouse cochlea peaked at P30-60, consistent with the time point of complete maturation of cochlear function (de Iriarte Rodriguez et al., 2015). Beginning at P365, the levels of these genes declined over time. Moreover, the results of cochlear immunofluorescence staining revealed that the autophagy marker LC3B was primarily localized on SGNs rather than on glial cells. The high-intensity labeling of autophagy markers in SGNs suggested that autophagy might play an important role in SGN development. Researchers found that ATG5 deficiency in auditory HCs could suppress autophagosome formation, which demonstrated that basal autophagy activity was impaired (Fujimoto et al., 2017). The ATG5^flox/flox^; Pou4f3-Cre mice exhibited accumulation of the autophagic substrate protein p62 and ubiquitinated proteins within HCs after 2 postnatal weeks. The auditory brainstem response (ABR) test results of ATG5-knockout mice at 4 or 8 postnatal weeks showed that at the frequencies of 2, 4, 16, and 32 kHz, the heterozygous mice displayed normal hearing whereas the homozygotes showed marked hearing loss. A morphological study demonstrated that the HC arrangements in the cochlea of homozygous mice were abnormal and hearing was severely impaired. The results from the studies above may indicate an essential role for autophagy in the process of cochlear development and functional maturation.

### Autophagy Protects Against Noise-Induced Hearing Loss (NIHL)

Some researchers have reported that in NIHL in CBA/J mice, the level of autophagy in permanent threshold shift (PTS) mice was lower than that in temporary threshold shift (TTS) mice, whereas the oxidative stress level in OHCs showed the opposite trend (Yuan et al., 2015). The oxidative stress markers 3-nitrotyrosine (3-NT) and 4-hydroxynonenal (4-HNE) in the OHCs of PTS mice noticeably declined after treatment with the autophagy agonist rapamycin. On the other hand, reduction of LC3B by the autophagy inhibitor 3MA or LC3B siRNA increased the levels of 3-NT in OHCs and promoted hair cell (HC) loss and NIHL. Therefore, according to this study, we consider that the level of autophagy in OHCs is universally elevated in NIHL mice, and the increased autophagic level presumably reduces the oxidative stress level of OHCs. Therefore, autophagy could ameliorate noise-induced OHC damage and hearing loss.

Heat shock proteins (HSPs) are a group of proteins that can assist in stabilizing newly synthesized polypeptides and correctly refolding damaged proteins. Among them, HSP70 possesses anti-stress and anti-apoptosis features (Mayer and Bukau, 2005). A study of mouse embryonic fibroblasts (MEFs) discovered that the level of acetylated HSP70 protein was upregulated following external stress. The acetylated HSP70 could not only bind to the Beclin1-Vps34 complex but also recruit KAP1 protein to SUMOylate Vps34 to enhance the formation of the Beclin1-Vps34 complex and finally to promote the formation of autophagosomes (Yang et al., 2013). Moreover, the Beclin1-Vps34 complex failed to develop after knockdown of the *HSP70* gene in MEFs, which was followed by decreased autophagosome formation (Park et al., 2008). Additionally, two large-scale gene screening programs in a noise-exposed population revealed that single nucleotide polymorphisms (SNPs) of HSP70, such as rs2227956 were associated with NIHL (Yang et al., 2006; Konings et al., 2009). It was reported that people with the C allele of SNP rs2227956 in the HSP70 gene was correlated with a remarkably increased level of serum HSP70 (Afzal et al., 2008). We assume that the HSP70 level in people with the C allele of rs2227956 is increased both in serum and in the inner ear. Therefore, in noise-exposure environments, a higher HSP70 level promotes autophagosome formation and leads to elevated efficiency of autophagy to remove noise-induced oxidative stress products, ultimately alleviating inner ear cell dysfunction and hearing loss.

### Autophagy Relieves Hearing Loss Induced by Ototoxic Drugs

Ototoxic drugs, including aminoglycoside antibiotics, cisplatin and loop diuretics, are the main causes of hearing loss in clinical practice. Most of these drugs damage the inner ear structure by elevating the level of oxidative stress. A recent study demonstrated that autophagic levels were significantly increased after neomycin or gentamicin administration in HC explants and HEI-OC1 cells (He et al., 2017). Furthermore, simultaneous treatment with rapamycin reduced aminoglycoside antibiotic-induced ROS levels and HC death, while treatment with the autophagy inhibitor 3-MA or deletion of the autophagy gene *ATG5* led to increases in ROS levels and cell apoptosis. Notably, the impairment of HC caused by 3-MA in HC explants could be efficiently blocked by NAC. Another study revealed that the levels of autophagy activity, OHC loss and the serum oxidative stress marker malonaldehyde (MDA), as well as the auditory threshold, were increased markedly after Wistar rats were injected with cisplatin (Fang and Xiao, 2014). Moreover, the autophagic level was further enhanced after treatment with rapamycin, accompanied by declines in OHC loss, auditory threshold and MDA levels. Based on these results, we speculate that the levels of oxidative stress and autophagy are increased in response to ototoxic drugs. Upregulating the level of autophagy by an autophagy activator may eliminate oxidative stress leading to protection of HCs, while suppressing autophagy via an autophagy inhibitor may increase oxidative stress leading to acceleration of HC death. Therefore, autophagy presumably functions as an efficient mechanism against oxidative stress (Giordano et al., 2014).

### Autophagy Impairment Contributes to Age-Related Hearing Loss

According to a WHO report, approximately 5.3% of the worldwide population (360 million) suffer from hearing disorders. Among elderly people aged above 65 years old, one-third have hearing impairment. Presbycusis, or ARHL, is a type of hearing loss resulting from interactions of physiological and environmental factors. Briefly, the fundamental pathology of ARHL is the degeneration of the auditory system. During aging, the capabilities of cell metabolism and self-repair progressively decline, leading to accumulations of abnormal or damaged proteins, lipids and organelles, which present as intracellular lipofuscin aggregations. These metabolic products are substrates that could be degraded by autophagy. In the development of the mouse inner ear, the level of autophagy continued to rise postnatally and did not decline until P365, which was coincident with the occurrence of ARHL (de Iriarte Rodriguez et al., 2015), indicating that autophagy dysfunction might exist in ARHL. The folding and assembly of proteins take place in the endoplasmic reticulum (ER). As one-third of all proteins might be incorrectly assembled during the synthesis process, the ER must perform quality control and degrade aberrant proteins. The unfolded protein response (UPR) is a cellular stress reaction that maintains the homeostasis of ER and protein folding, facilitating normal folding and directly degenerating misfolded proteins. Some researchers have reported that the UPR was impaired in the cochlea of aged C57BL/6 mice (12–14 months), and the level of ubiquitinated proteins was obviously higher than in young mice (1–2 months), indicating the existence of accumulated aberrant proteins and increased ubiquitinated proteins in degenerated cochlea (Wang et al., 2015). A study also found that compared with antisenescence SAMR1 mice, the IHCs and OHCs in the cochlea of pro-senescence SAMP8 mice were severely damaged, and products of oxidative stress, such as 8-oxoG, were distinctly increased in HCs during aging (1–12 months) (Menardo et al., 2012). Meanwhile, the accumulation of lipofuscin in SGNs occurred at a significantly higher rate in SAMP8 mice than in SAMR1 mice. Lipofuscin, the remnant of autolysosomes, is a highly cross-linked and undegradable aggregate. The progressive accumulation of lipofuscin and ubiquitinated proteins convincingly demonstrated that autophagy was impaired in degenerated SGNs. A recent study reported that the levels of LC3 and the substrate protein p62 were increased in the cochlea of aged C57BL/6J mice, which also confirmed the phenomenon of impaired autophagy in ARHL mice (Pang et al., 2017). In addition, it was reported that the expression of miR-34a, which is associated with ARHL, was also increased in the aging cochlea. In HEI-OC1 cells, overexpression of miR-34a resulted in autophagy flux impairment and cell activity suppression, which could be counteracted by miR-34a inhibition. Further analysis showed that *ATG9a*, one of the indispensable genes involved in the formation of autophagosomes and the fusion of autophagosomes with lysosomes, acted as a target gene of miR-34a. Therefore, we presume that in the cochlea of aging mice, the downregulation of ATG9a caused by progressively elevated expression of miR-34a leads to accumulation of autophagosomes and deficient autophagosome-lysosome fusion, resulting in autophagy impairment and cell death. These findings demonstrate the existence of abnormal autophagic mechanisms in ARHL mice. Notably, impaired autophagy has proved to be proved a critical factor involved in a variety of neurodegenerative disorders, such as Alzheimer’s disease, Parkinson’s disease, and amyotrophic lateral sclerosis (Menzies et al., 2015). In summary, many researchers have reported that autophagy upregulation could have a protective role in specific hearing loss such as ototoxicity drugs, noise and aging (Table 1).

**Table 1:** Overview the role of autophagy mechanism *in vitro* and *in vivo* models about hearing.

Model	Disease model	Drug / Target	Dose	Sample Size	Hearing threshold shift	Histological observations	Autophagy mechanism	Reference
C57BL/6 mice (8W)	Atg5^-/-^ in HCs	/	/	14	80–90 dB↑ (8,16 kHZ)	HCs damaged	Autophagosome formation impaired	Fujimoto et al., 2017
CBA/J mice (12W)	NIHL (TTS)	3-MA	30 mg/kg	6	25 dB ↑ (16 kHZ)	OHCs loss severely	Autophagy level decreased	Yuan et al., 2015
CBA/J mice (12W)	NIHL (PTS)	Rapamycin	7.5 mg/kg	14	15 dB↓ (16 kHZ)	Remained OHCs increased	Autophagy level upregulated	Yuan et al., 2015
HEI-OC1 cells	Neomycin	3-MA	5 mM for 6 h	/	/	TUNEL positive cells increased	Autophagy level decreased	He et al., 2017
HEI-OC1 cells	Neomycin	Rapamycin	0.1 mM for 6 h	/	/	TUNEL positive cells decreased	Autophagy level upregulated	He et al., 2017
Wistar rats (8w)	Cisplatin	Rapamycin	2 mg/Kg	8	20dB↓ (16kHZ)	Alleviated HC damage	Autophagy level upregulated	Fang and Xiao, 2014
HEI-OC1 cells	ARHL	^Si-ATG9a^	20 μM	/	/	Survival cells decreased	Autophagic flux inhibited	Pang et al., 2017


### Mutations in Autophagy-Related Genes Contributes to Hereditary Hearing Loss

Sensorineural hearing loss can be caused by environmental and hereditary factors, and approximately 60% of cases result from the latter. Many deafness genes have crucial roles in autophagy. Although the three types of autophagy vary in initiation signals and degradation substrates, their degradation site is the same: the lysosome. The existence of the same end path for different types of autophagy makes lysosomal function important (Shen and Mizushima, 2014). Normal lysosomal function depends on the activity and number of proteolytic enzymes and the maintenance of the acidic microenvironment. Lysosomal storage disorders (LSDs) are a series of metabolic disorders that are mainly due to mutations in genes encoding proteolytic enzymes within lysosomes, resulting in the aggregation of relevant substrates and cell death (Lieberman et al., 2012). Abnormal hearing function has already been observed in several types of LSD, such as Gaucher disease (mutations in the β-glucosidase gene) (Zhao et al., 2003), Fabry disease (mutations in the α-galactosidase A gene) (Suntjens et al., 2015), Pompe disease (mutations in the acid α-glucosidase gene) (van Capelle et al., 2010), and β-mannosidosis (mutations in the β-mannosidosis gene) (Dorland et al., 1988). The maintenance of the acidic microenvironment requires delivering H^+^ into the lysosomes by the H^+^-ATPase pump. Among them, an important type is the vacuolar ATPase (v-ATPase) which is constituted by subunits such as ATP6V1B1, ATPV1B2 and ATP6V0A4. DDOD (dominant deafness-onychodystrophy syndrome) is an autosomal dominant syndromic deafness characterized by nonsense mutations of ATP6V1B2 (Yuan et al., 2014). ATP6V1B1 and ATP6V0A4 are the major pathogenic genes related to distal renal tubular acidosis (dRTA), a hereditary disease distinguished by metabolic acidosis, severe hyperchloremia, impaired bone growth and SNHL (Stover et al., 2002). Mutations in these genes are certain to cause lysosomal dysfunction. Theoretically, these genetic mutations may lead to lysosomal damage, resulting in autophagy dysfunction by suppressing the degradation of autophagosomes in lysosomes and finally threatening cell survival.

miR-96 is the first miRNA mutation reported to be associated with human deafness, which are seed region mutations which result in non-syndromic SNHL (Mencia et al., 2009). A study revealed that in ethylnitrosourea (ENU)-induced miR-96 mutant mice, the heterozygotes displayed progressive hearing loss, while the homozygotes showed no cochlear response (Lewis et al., 2009). However, although miRNA is an essential factor for regulating mRNA levels, the genes downstream of miR-96 in cochlea remain unclear. Recent studies on tumor (Yu et al., 2018) and brain injury (Gan et al., 2017) suggested that *ATG7* could be modulated by miR-96. As a ubiquitin-like ligase, ATG7 could activate ATG12 and ATG8, both of which are essential for the formation of autophagosomes (Hong et al., 2011). According to one study, reduced miR-96 expression, owing to its mutation, may directly upregulate the level of *ATG7*, which leads to increased synthesis of autophagosomes followed by hyperactivated autophagy. The hyperactivation of autophagy ultimately results in neuronal degeneration and death (Bandyopadhyay et al., 2014).

## Autophagy Is an Efficient Antioxidant Pathway in Inner Ear Disorders

Recent studies on the role of the autophagic mechanism in the auditory system have mainly focused on using the antioxidant capacity of autophagy to protect HCs and hearing (Fang and Xiao, 2014; Yuan et al., 2015; He et al., 2017). Oxidative stress can be observed in hearing loss induced by many exogenous factors, such as noise (Yuan et al., 2015), ototoxic drugs (He et al., 2017) and radioactive radiation (Tan et al., 2013), as well as by intrinsic factors, such as ischemia (Maetani et al., 2003) and aging (Fujimoto and Yamasoba, 2014). Oxidative stress indicates that the increased intracellular free radicals and ROS induced by injuries exceed the protective capacity of the antioxidant system and therefore fail to be effectively eliminated, eventually leading to cell damage and death (Finkel and Holbrook, 2000; Lechowicz et al., 2018). Although there is still controversy about whether inflammation, autophagy or dystrophy is involved in the development of SNHL, many researchers agree that oxidative stress is a key mechanism for SNHL development. Many antioxidants, such as ebselen (Kil et al., 2017), NAC (Yuan et al., 2015), and L-cysteine (Lu et al., 2011), have been applied to treat SNHL in experimental studies, and the results demonstrated that antioxidant agents showed potential for treating SNHL. Glutathione peroxidase 1 (GPX1), the main antioxidant enzyme in cells, constitutes the antioxidant system along with intracellular superoxide dismutase (SOD), catalase and other enzymes. There are four forms of GPX. Among them, GPX2-GPX4 were expressed at low levels or were undetectable in the inner ear, while GPX1 was highly expressed in HCs, supporting cells, the stria vascularis and spiral ligaments, especially in SGNs. Nearly two decades ago, researchers reported that adult GPX1-knockout mice exposed to broadband noise showed an elevated ABR threshold and severely damaged HCs when compared with wild-type mice (Ohlemiller et al., 2000). Furthermore, that study also found that treatment with ebselen could prevent mice from NIHL and efficiently alleviate the swelling of afferent dendrites following IHC and SGN loss. Ebselen is the most effective antioxidative molecule containing organic selenium that mimics GPX1 activity. A recent study in *Lancet* in 2017 reported a randomized double-blind phase II trial examining ebselen treatment of NIHL patients (Kil et al., 2017). According to their research, taking 400 mg ebselen twice a day could prevent temporary auditory threshold shifts caused by acute noise exposure. We think that oxidative stress may be more pronounced in the occurrence and development of NIHL. The important features of aging in mammals are the progressive decline in mitochondrial oxidative phosphorylation and the continuous accumulation of ROS. Because mitochondria produce 90% of the cellular ROS, they are also the main target of ROS (Fujimoto and Yamasoba, 2014).

Mitochondrial DNA (mtDNA) is located in the mitochondria and is vulnerable to stress in the absence of a robust DNA repair mechanism. Increased ROS, which are also synthesized in the inner membrane of mitochondria, can attack mtDNA, resulting in mutations or loss of large fragments, accompanied by accumulation products of oxidative stress damaging to mtDNA such as 8-OhdG (Hamalainen et al., 2015). Damage to mtDNA in HCs, supporting cells, SGNs and other cells would further influence the function of the mitochondrial antioxidant system in the inner ear and increase the synthesis of ROS, ultimately forming a vicious cycle of ROS and mtDNA damage. The level of the oxidative product MDA in serum from pro-senescence SAMP8 mice at 1 month was 2–3 times than that in serum from antisenescence SAMR1 mice at 9 months, and the SOD1 activity in the inner ear was decreased by 40–50% in SAMP8 mice at 9 months (Menardo et al., 2012). Moreover, in the same study they examined the fluorescence intensity of 8-OHdG in the cochlea and found that the somas of HCs and SGNs in SAMP8 mice at 1 month were clearly stained, but no 8-OHdG staining was observed in SAMR1 mice at 9 months. Taking these results together, oxidative stress apparently contributes to the pathological changes observed in SNHL. Therefore, antioxidant treatment may be a potential method to treat hearing loss.

**FIGURE 1:**
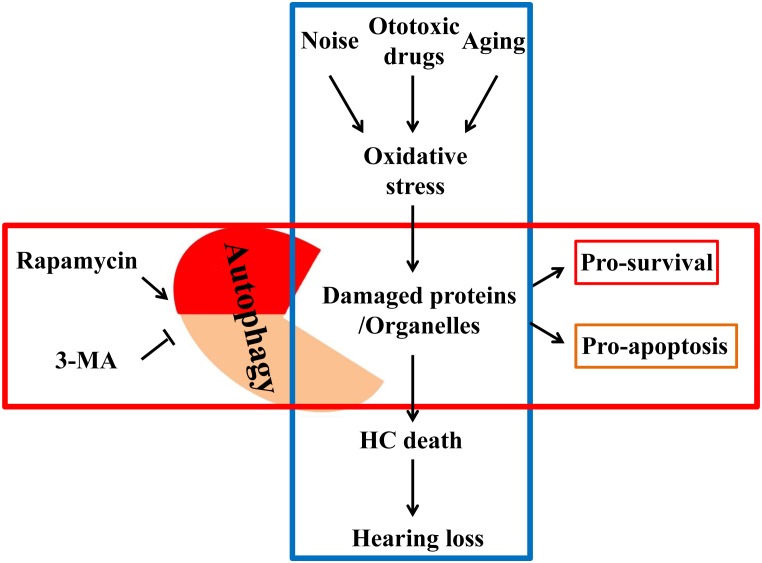
External factors such as noise, ototoxic drugs and aging elevate the level of oxidative stress in the inner ear. Elevated oxidative stress can deteriorate proteins and organelles like mitochondria, ultimately leading to hair cell death and hearing loss. Autophagy can phagocytose these oxidative products for reuse to promote cell survival by autophagy regulators, so that cells can be in a healthy state.

In the past, antioxidative therapy has always concentrated on reducing ROS release or enhancing intracellular antioxidant capacity. However, for already oxidized proteins or damaged organelles, conventional antioxidants often remain limited in their effectiveness (Sies, 2015). Understanding autophagic mechanisms leads to the realization that autophagy can not only remove damaged, oxidized lipids and proteins but also can remove impaired mitochondria (the main site of ROS production). Therefore, autophagy can protect the cells by degrading the already oxidized proteins and ROS. In short, autophagy not only eliminates free radicals or ROS but also directly removes damaged mitochondria, which continuously release ROS, making it an ideal means of anti-oxidation (Giordano et al., 2014). A previous study reported that *ATG5* knockout in mice caused neurodegeneration in the brain. The pathological analysis revealed that massive inclusion bodies composed of peroxidase proteins and lipids had accumulated in the mouse brain, primarily in neurons (Hara et al., 2006). Many *in vivo* and *in vitro* experiments have confirmed that autophagy is one of the basic antioxidant pathways. Increasing the level of autophagy could reduce oxidative stress, thus effectively protecting cells, especially in neurodegeneration. Hence, appropriately elevating the level of autophagy could prevent SNHL induced by ototoxic drugs, noise and aging (Figure 1).

## Open Questions and Future Directions

Taking all these findings into consideration, we believe that autophagy plays a crucial role in hearing loss induced by extrinsic stimuli or intrinsic genetic mutations. However, research thus far only seems to focus on manipulating the autophagy level and mainly considers autophagy as an efficient tool against oxidative stress. The underlying mechanisms of autophagy and its related molecules/signaling pathways in SNHL remain largely unrevealed. What specific molecules are involved in hearing loss? Autophagic dysfunction occurs in many neurodegenerative diseases and neuronal injuries (Nixon, 2006); does it, then, exist in inner ear damage? Upregulating autophagy was beneficial for hearing preservation, but hyperactivated autophagy can also promote cell death. Therefore, precisely manipulating the autophagy level in various models of hearing pathology still needs to be further explored. In conclusion, further investigating the mechanism of autophagy will facilitate its utilization in the protection of cochlear cells and auditory capacity.

## Author Contributions

MX: substantial contributions to the design of the work. BY, CF, YS, QW, and HH: drafting the work or revising it critically for important intellectual content, the authors agree to be accountable for all aspects of the work in ensuring that questions related to the accuracy or integrity of any part of the work are appropriately investigated and resolved. MX: approval of the final version of this article.

## Conflict of Interest Statement

The authors declare that the research was conducted in the absence of any commercial or financial relationships that could be construed as a potential conflict of interest.

## Abbreviations

ARHL: age-related hearing loss
ROS: reactive oxygen species
SAMP8: senescence accelerated mouse prone 8
SAMR1: senescence accelerated mouse resistance 1
SNHL: sensorineural hearing loss
